# Impact of chronic intermittent hypoxia on the long non‐coding RNA and mRNA expression profiles in myocardial infarction

**DOI:** 10.1111/jcmm.16097

**Published:** 2020-11-20

**Authors:** Chaowei Hu, Jing Li, Yunhui Du, Juan Li, Yunyun Yang, Yifan Jia, Lu Peng, Yanwen Qin, Yongxiang Wei

**Affiliations:** ^1^ Key Laboratory of Upper Airway Dysfunction‐related Cardiovascular Diseases Beijing Institute of Heart, Lung and Blood Vessel Diseases Beijing Anzhen Hospital Capital Medical University Beijing China; ^2^ Heart Center & Beijing Key Laboratory of Hypertension Beijing Chaoyang Hospital Capital Medical University Beijing China; ^3^ Key Laboratory of Remodeling‐related Cardiovascular Diseases Beijing Institute of Heart, Lung and Blood Vessel Diseases Beijing Anzhen Hospital Capital Medical University Beijing China; ^4^ Department of Cardiology Beijing Anzhen Hospital Capital Medical University Beijing China; ^5^ Otolaryngological Department of Beijing Anzhen Hospital Capital Medical University Beijing China

**Keywords:** chronic intermittent hypoxia, lncRNA, mRNA, myocardial infarction

## Abstract

Chronic intermittent hypoxia (CIH) is the primary feature of obstructive sleep apnoea (OSA), a crucial risk factor for cardiovascular diseases. Long non‐coding RNAs (lncRNAs) in myocardial infarction (MI) pathogenesis have drawn considerable attention. However, whether CIH participates in the modulation of lncRNA profiles during MI is yet unclear. To investigate the influence of CIH on MI, cardiac damage was assessed by histology and echocardiography, and lncRNA and mRNA integrated microarrays were screened. MI mouse model showed myocardial hypertrophy, aggravated inflammation and fibrosis, and compromised left ventricle function under CIH. Compared with normoxia, 644 lncRNAs and 1084 differentially expressed mRNAs were identified following CIH for 4 weeks, whereas 1482 lncRNAs and 990 mRNAs were altered at 8 weeks. Strikingly, reoxygenation after CIH markedly affected 1759 lncRNAs and 778 mRNAs. Of these, 11 lncRNAs modulated by CIH were restored after reoxygenation and were validated by qPCR. The GO terms and KEGG pathways of genes varied significantly by CIH. lncRNA‐mRNA correlation further showed that lncRNAs, *NONMMUT032513* and *NONMMUT074571* were positively correlated with ZEB1 and negatively correlated with Cmbl. The current results demonstrated a causal correlation between CIH and lncRNA alternations during MI, suggesting that lncRNAs might be responsible for MI aggravation under CIH.

## 1 INTRODUCTION

Myocardial infarction (MI) results from acute coronary artery stenosis and occlusion and is often complicated with arrhythmia and heart failure, leading to high mortality.^1^ The cardiac remodelling after MI is associated with inflammation, fibrosis and cardiomyocyte apoptosis.^2^ The lack of oxygen supply during MI causes acute, massive cardiac cell death within minutes.^3^

Obstructive sleep apnoea (OSA) is a common sleep disorder in the adult population and is associated with various cardiovascular diseases. It has been recognized as an independent risk factor for hypertension, arrhythmia and heart failure, among other conditions.^4^, ^5^ Chronic intermittent hypoxia (CIH) results from the collapse of upper airways and is a characteristic and the primary pathogenic factor of OSA.^6^ Accumulating evidence suggested that CIH plays a major role in the process of MI.^7^, ^8^, ^9^ It also leads to enhanced infarct area via increased ROS levels or modulation of endoplasmic reticulum stress in cardiomyocytes.^10^, ^11^

Non‐coding RNAs without protein‐coding potential, including long non‐coding RNAs (lncRNAs, >200 nucleotides) and microRNAs (miRNAs, <200 nucleotides), play critical roles in the pathophysiology of cardiovascular diseases, including MI. However, some studies suggested that lncRNAs display various biological functions^12^ that include indirect regulation of gene expression. In turn, these phenomena affect cellular processes via post‐transcriptional regulation and nuclear transport.^13^, ^14^ Recently, the potential molecular mechanisms of lncRNAs in cardiovascular diseases have gained increasing attention. lncRNAs *LIPCAR* and *UCA1* have been identified as the biomarkers of heart failure and acute MI, respectively.^15^, ^16^, ^17^, ^18^

However, the effect of CIH on the expression profiles of lncRNA and mRNA in MI mice and whether specific lncRNAs are critical for MI pathogenesis remains to be elucidated. Therefore, the present study aimed to confirm the causal correlation between CIH and cardiac damage after MI and explore the lncRNA expression profiles.

## 2 MATERIALS AND METHODS

### 2.1 Animal model

C57BL/6J male mice, 8‐week‐old, were randomly divided into MI and sham groups. Surgical MI was induced by ligation of the left anterior descending coronary artery. Sham‐procedure mice underwent the same protocol but without ligation of the coronary artery. Then, the mice were randomly subjected to normoxia and intermittent hypoxia (5% O_2_ at nadir, 20 cycles/h) for 4 or 8 weeks, as described previously.^19^ At the end of the exposure, the animals were killed after anaesthesia with 2% isoflurane. The hearts were collected for the quantitation of lncRNA and mRNA expression profiles and histology. The research protocol was approved by the Animal Care and Use Committee of Capital Medical University, and the subsequent animal experiments were performed based on the guidelines of the Animal Ethics Committee of the University.

### 2.2 Histology

Heart tissues were fixed with 4% paraformaldehyde, embedded in paraffin and sectioned, followed by staining with haematoxylin‐eosin (HE) to determine the severity of myocardial inflammation. The collagen volume at the free wall of the infarct area was assessed by Masson's staining (n = 6/group). For Masson's trichrome, power analysis shows that the sample size of 6/group has a 91%~99% power assuming a 5% significance level with a two‐sided test.

For immunohistochemistry, the paraffin sections of the infarcted heart were incubated with vWF antibody (Proteintech, 66682‐1) and CD31 antibody (BD Pharmingen™, 553370) overnight at 4°C to determine the capillary density and with α‐smooth muscle actin (α‐SMA) antibody (Beyotime Biotechnology, AA132) to examine the arteriole density. Followed by the incubation of secondary antibodies at room temperature for 1 hour, the sections were stained with avidin‐biotin complex and counterstained with haematoxylin. The images were captured under a microscope (Nikon), and the capillary and arteriolar density was calculated as the number of capillaries or arterioles/mm^2^ (n = 4‐5/group). For immunohistochemistry staining, power analysis shows that the sample size of 4‐5/group has a 100% power assuming a 5% significance level with a two‐sided test.

### 2.3 Echocardiography

The animals were anaesthetized with 2% isoflurane during echocardiography and examined on a VisualSonics Vevo 2100 system using a 30 MHz‐Transducer (MS‐400; VisualSonics). The left ventricular wall thickness was analysed using M‐mode images from the parasternal short‐axis view. n = 8 for Sham + Air (4 weeks), n = 7 for Sham + CIH (4 weeks), n = 6 for MI + Air (4 weeks), n = 8 for MI + CIH (4 weeks), n = 4 for MI + Air (8 weeks) and n = 4 for MI + CIH (8 weeks). For echocardiography, power analysis shows that the sample size of 4~8/group has a 86%~100% power assuming a 5% significance level with a two‐sided test.

### 2.4 Western blot

Tissues were homogenized with RIPA buffer containing protease inhibitors (Applygen Technologies Inc), and proteins in the supernatants were extracted following centrifugation. The concentration of protein was determined by the BCA Protein Concentration Assay Kit (Boster). Thirty micrograms protein samples were electrophoresed and fractionated on 8% SDS‐PAGE, and transferred onto nitrocellulose membranes (Millipore). The membranes were blocked with 5% non‐fat milk in Tris‐buffered saline buffer (20 mmol/L Tris, 150 mmol/L NaCl, pH 7.6 Tween‐20 0.1%) and incubated with HIF‐1α (Abcam, ab82832) or β‐actin (ZSGB‐BIO, TA‐09) primary antibodies respectively at 4°C overnight. Followed by binding with specific secondary antibodies, the protein blots were detected with enhanced chemiluminescence reagents. β‐actin served as an endogenous control. The band signal intensities were quantitatively analysed by Quantity One software, n = 4 for each group. For western blot, power analysis shows that the sample size of 4/group has a 100% power to detect a difference of 0.2~0.3, assuming a 5% significance level with a two‐sided test.

### 2.5 RNA preparation, microarray processing and analysis

Total RNA was extracted using TRIzol reagent and quantified using NanoDrop ND‐2000. RNA integrity was assessed using Agilent Bioanalyzer 2100. Clariom™ D assays (Applied Biosystems) were employed to profile lncRNA and mRNA expression. Total RNA was transcribed to cDNA, synthesized into cRNA and labelled with cyanine‐3‐CTP, followed by hybridization onto the microarray. Following a wash step, the arrays were scanned using Agilent Scanner G2505C (Agilent Technologies). A random variance model (RVM) *t* test was applied to filter the differentially expressed genes according to the *P‐*value threshold. Hierarchical clustering was employed to analysed the differentially expressed lncRNA and mRNA (n = 4/group).

### 2.6 Quantitative PCR (qPCR)

qPCR was performed to validate the expression of significantly altered lncRNA in heart tissues from the mouse model. Total RNA was extracted using TRIzol reagent (Invitrogen) and purified with an RNeasy kit (Qiagen). Total RNA was reverse‐transcribed using M‐MLV reverse transcription kit (Promega), according to the manufacturer's instructions. qPCR analysis and data collection were performed on the ABI 7500 Real‐Time PCR System (Applied Biosystems) using the specific primer pairs (Table S1). *GAPDH* served as an endogenous control for normalization of the expression of each target gene. 2^−ΔΔCt^ was calculated to indicate the relative expression of the gene. For quantitative PCR, power analysis shows that the sample size of 3/group has a 91%~96% power assuming a 5% significance level with a two‐sided test.

### 2.7 Gene ontology (GO) and Kyoto Encyclopedia of Genes and Genomes (KEGG) analysis

GO analysis and KEGG pathway analysis were applied to identify the significant functions and pathways of the differentially expressed mRNA. A two‐sided Fisher's exact test and chi‐square test were used to classify the GO category, and *P*‐values were corrected for multiple comparisons by calculating the false discovery rate (FDR). Pathway analysis was conducted according to KEGG, Biocarta and Reactome. Two‐sided Fisher's exact test and chi‐square test were used to select the significant pathways with the threshold of significance defined by FDR‐corrected *P*‐values.

### 2.8 Gene‐gene functional interaction network

An interaction network was constructed based on the data for differentially expressed genes. The KEGG database was used to analyse the functional gene interactions, and Cytoscape software was used to build the network. In the network, each gene corresponded to a node, and the nodes were connected by an edge. The degree of the gene expression was defined as the number of directly linked genes within a network, which could assess the relative significance of a gene to the network. Thus, a gene that connects to a large number of adjacent genes in the network is vital for the network. In addition, the genes were also analysed for betweenness centrality, an indicator of the gene centrality in a specific network. The betweenness centrality is equal to the number of shortest paths from all vertices to the others that pass through a gene. Thus, the degree and betweenness centrality were used as two indicators to identify the importance of a specific gene.

### 2.9 lncRNA‐mRNA expression correlation network

A lncRNA‐mRNA expression correlation network was built based on the normalized signal intensity of the specific expression in mRNA and lncRNA. Pearson's correlation was used to choose significant mRNA‐lncRNA, mRNA‐mRNA or lncRNA‐lncRNA correlation pairs, and the cut‐off of the correlation value was set at .92. The centrality of a gene or lncRNA was detected within the network. While considering different networks, core genes were determined based on the degree differences between two group samples.

### 2.10 Statistical analysis

Student's *t* test was used for data comparison between the two groups, and the statistical differences among the groups were evaluated by one‐way analysis of variance (ANOVA) or the Kruskal‐Wallis test. *P*‐values < .05 indicated significant difference. Data are presented as mean ± standard error of the mean (SEM).

## 3 RESULTS

### 3.1 CIH aggravates cardiac injury after MI

**Figure 1:**
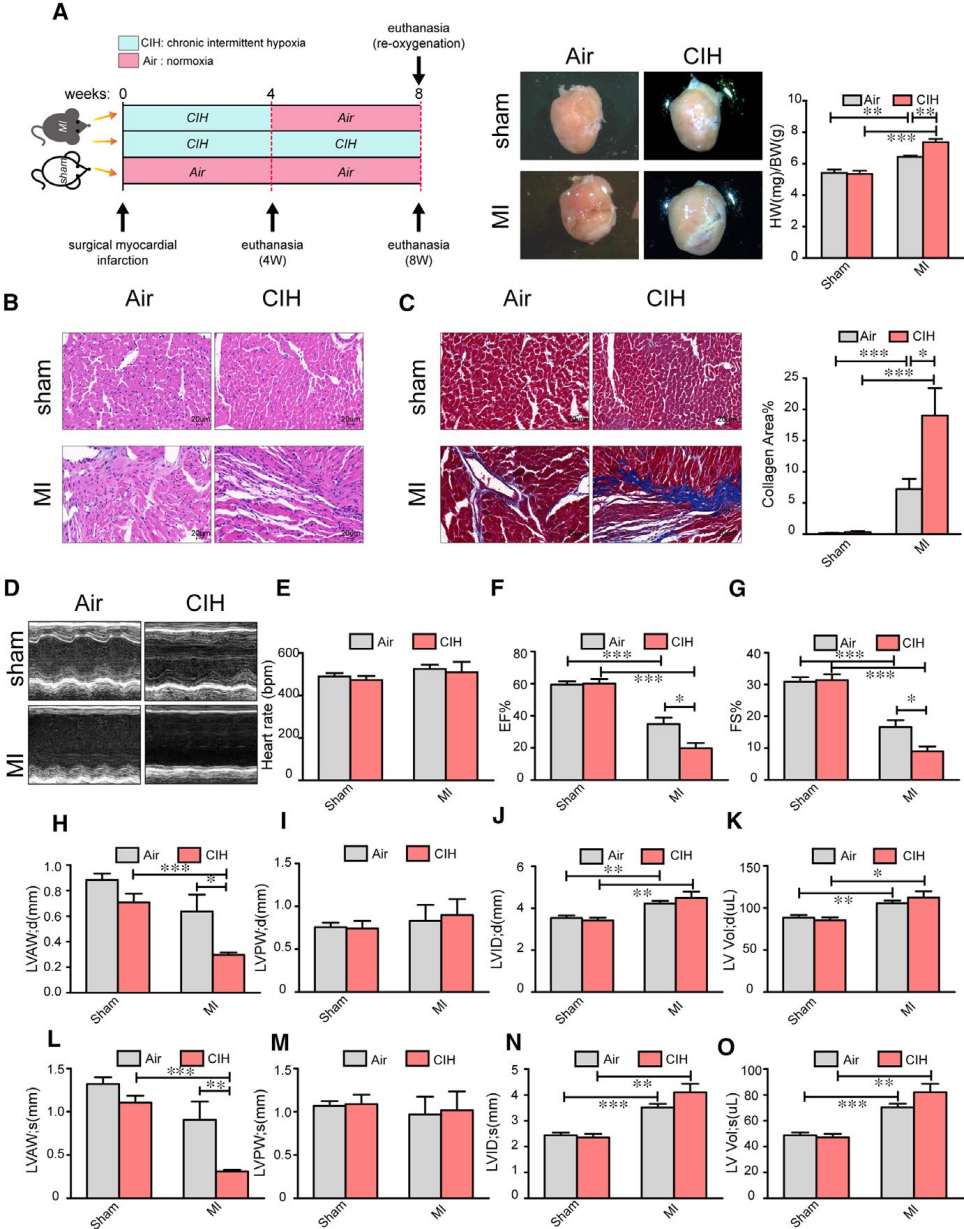
CIH modulates cardiac injury post‐infarction. A, Experimental scheme for establishing the mouse model. A representative whole heart image for the quantification of cardiac hypertrophy burden. HW/BW indicates the heart weight/bodyweight. n = 11 for Sham + Air, n = 10 for Sham + CIH, n = 7 for MI + Air and n = 8 for MI + CIH. B and C, Cross sections of heart tissues were stained by HE and Masson, respectively, and cardiomyocyte inflammation and cardiac fibrosis were determined. n = 6 for each group. D, Cardiac function was examined by echocardiography, and representative left ventricular M‐mode echocardiography images are shown. E‐O, Quantification of heart rate, EF, FS, LVAWd (left ventricular diastolic anterior wall), LVAWs (left ventricular systolic anterior wall), LVPWd (left ventricular diastolic posterior wall), LVPWs (left ventricular systolic posterior wall), LVIDd (left ventricular internal diastolic diameter), LVIDs (left ventricular internal systolic diameter), LV Vol‐d (left ventricular diastolic volume) and LV Vol‐s (left ventricular systolic volume). Air indicates normoxia. n = 8 for Sham + Air, n = 7 for Sham + CIH, n = 6 for MI + Air and n = 8 for MI + CIH. *P < .05, **P < .01, ***P < .001. Data are presented as mean ± SEM

Cardiac damage is a hallmark of MI. To determine the effects of CIH on cardiac remodelling post‐infarction, we exposed male mice with MI surgery to a CIH environment (5% O_2_ at nadir, 20 cycles/h) for 4 and 8 weeks to mimic OSA conditions, as described previously.^19^ Normoxia‐exposed sham‐surgery mice served as controls. To investigate whether CIH modulates the cardiac remodelling post‐infarction, we measured heart weight (HW) and analysed the cardiac function by echocardiography. MI mice subjected to CIH exhibited a high cardiac hypertrophy index, as indicated by the HW/bodyweight (BW) ratios at 4 weeks (Figure 1A). Following 8‐week CIH, the HW/BW ratios of MI mice have reached a threshold, with no difference between Air and CIH (Figure S2A). The analysis of heart morphology revealed exacerbation of pathological remodelling post‐infarction by CIH treatment, with an increased incidence of heart rupture, aggravated local inflammation and severe myocardial fibrosis in MI mice exposed to CIH (Figure 1B,C). Myocardial remodelling was further confirmed by measuring the capillary and arteriolar density. As shown in Figure S1A‐C, both capillary (vWF and CD31 marked) and arteriolar (α‐SMA marked) densities were significantly reduced in MI hearts as compared to the sham controls, and CIH exposure led to suppressed myocardial arteriolar density after MI. MI‐induced cardiac dysfunction was greater in the 4‐week CIH group based on decreased left ventricular ejection fraction (LVEF), fractional shortening (FS) and left ventricular anterior wall (LVAW) (Figure 1D‐O), which was not significant following 8‐week CIH (Figure S2B‐M). MI‐induced cardiac dysfunction was great in both the 8‐week CIH and Air group, with quite low EF and FS (Figure S2D,E). Additionally, myocardial HIF‐1α expression was measured to examine the effect of CIH exposure. It was observed that compared with normoxia, CIH was conducive to high mRNA and protein expression of HIF‐1α in the infarcted heart (Figure S1D,E). These findings demonstrated that exposure to CIH worsens the heart injury post‐MI.

### 3.2 lncRNA and mRNA expression profiles under MI are affected by CIH

lncRNAs have been considered as critical players in cardiovascular disease by interacting with proteins and other RNAs to regulate the downstream gene expression. To explore the mechanism of CIH in worsening cardiac injury and assess how CIH modulates the expression profile of lncRNA and mRNA during MI, cardiac lncRNA and mRNA expression profiles were identified by Clariom™ D assays (mouse). The lncRNA and mRNA with *P* ≤ .05 (*t* test) and fold change expression ≥ 1.2 were regarded as differentially expressed between the groups.

**Figure 2:**
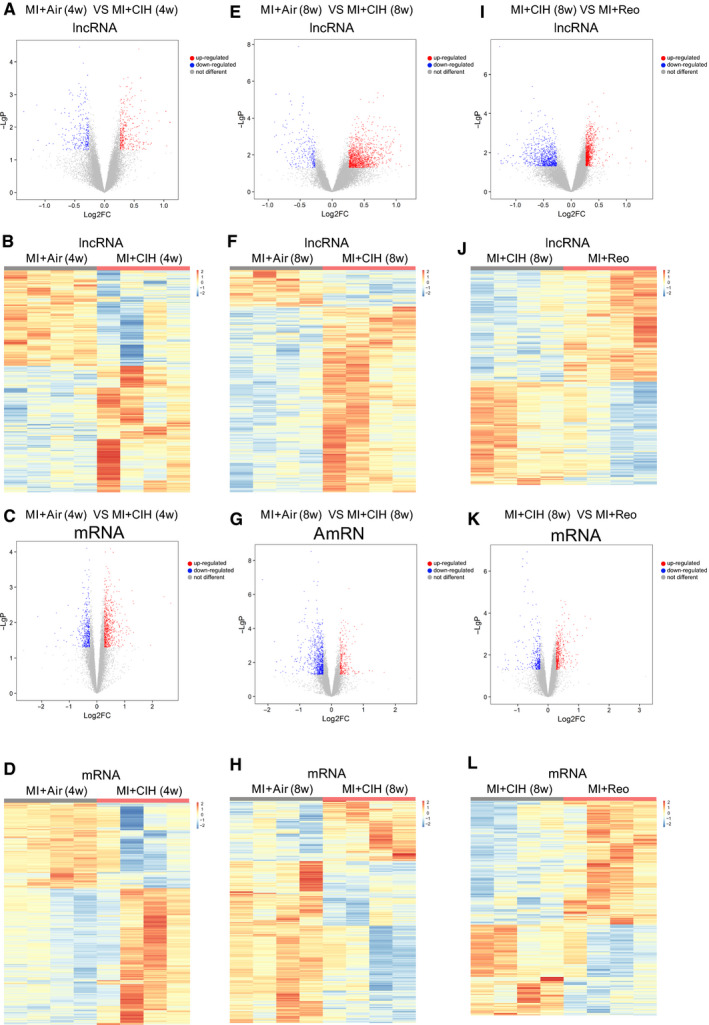
Specific lncRNAs and mRNAs regulated by CIH and reoxygenation under MI. A and C, Volcano plots showing the lncRNAs and mRNAs significantly regulated by 4‐week exposure to CIH. The lncRNAs and mRNAs with *P ≤ *.05 (*t* test) and fold change in expression ≥ 1.2 were regarded as differentially expressed between the groups. B and D, Heat map depicting the changes in lncRNAs and mRNAs in MI mice after exposure to CIH for 4 wk. E and G, Volcano plots of the significantly different lncRNAs and mRNAs between MI and MI + CIH group at 8 wk. F and H, Heat map of the relative abundance of significantly changed lncRNAs and mRNAs by CIH at 8 wk. I and K, The lncRNAs and mRNAs that varied between MI complicated with CIH (8 wk) and MI with reoxygenation (4 wk) after CIH (4 wk) are shown in the volcano plots. J and L, The relative abundance of lncRNAs and mRNAs in I and K is shown in the heat map. Air indicates normoxia; Reo indicates reoxygenation after CIH; 4W indicates 4 wk; and 8W indicates 8 wk. The red scatters in volcano plots indicate genes up‐regulated by CIH, blue scatters indicate genes down‐regulated, and grey scatters indicate genes that are not different between the groups. The heat map scale indicates the relative abundance of specific genes that were transformed into *Z* scores. The value of (−log P) was the base 10e negative logarithm of the *P*‐value. n = 4/group

After 4 weeks of CIH exposure, 644 lncRNAs and 1084 mRNAs were identified as differentially expressed in MI mice as compared to those subject to normoxia, with 366 lncRNAs increased and 278 lncRNAs reduced, 668 mRNAs increased and 416 mRNAs reduced (Figure 2A,C). After an 8‐week exposure time to CIH, 1482 lncRNAs and 990 mRNAs were significantly altered, including 1235 up‐regulated and 247 down‐regulated lncRNAs, and 270 increased mRNAs and 720 decreased mRNAs (Figure 2E,G). The relative abundance of the lncRNAs and mRNAs affected by CIH under MI injury is shown in Figure 2B,D,F,H. In sham mice, 1462 lncRNAs and 523 mRNAs were identified as significantly differentially expressed after 4 weeks of CIH exposure as compared to those subject to normoxia (Figure S3A‐D). After 8‐week exposure to CIH, 360 lncRNAs and 265 mRNAs were altered significantly (Figure S3E‐H). We further analysed the overlap between the CIH‐induced differential expression of lncRNA and mRNA in Sham and MI conditions. There were 40 lncRNAs and 47 mRNAs altered by 4‐week CIH in both Sham and MI conditions, 31 lncRNAs and 40 mRNAs shift under 8 weeks CIH simultaneously in Sham and MI mice. We also detected 604 lncRNA and 1037 mRNA varied following 4 weeks CIH exclusively within a MI background, 1451 lncRNA and 950 mRNA altered by 8 weeks CIH exclusively in MI mice (Figure S4). Thus, the global lncRNA and mRNA expression profiles of MI are markedly modified by CIH intervention. To further explore the impact of CIH, an additional reoxygenation group was included, and the lncRNA and mRNA expression profiles in MI mice with reoxygenation after CIH were distinctly different from those without reoxygenation. A total of 1759 lncRNA and 778 mRNAs with significantly altered levels were identified, including 914 up‐regulated and 845 down‐regulated lncRNAs, 450 mRNAs increased and 328 mRNAs reduced (Figure 2I,K). The relative abundance of these differentially expressed lncRNAs is shown in Figure 2J.

**Figure 3:**
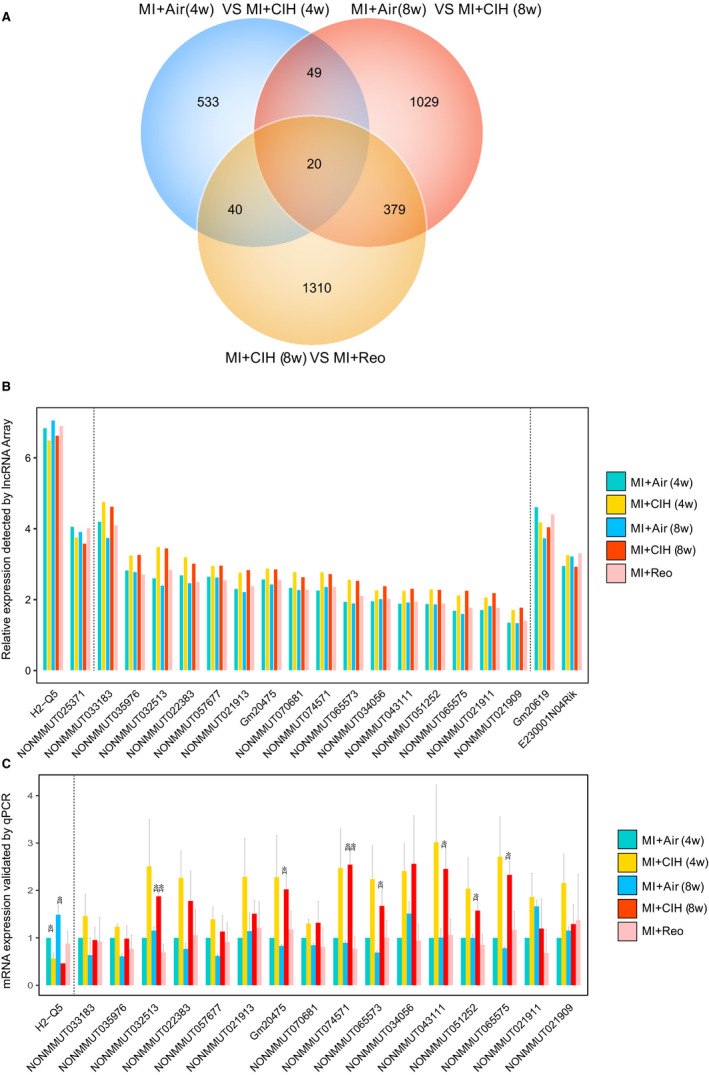
lncRNAs responsive to CIH post‐infarction. A, Venn diagram showing the number of lncRNAs that are statistically altered by 4 and 8 wk of CIH post‐MI, as well as the lncRNAs regulated by reoxygenation (4 wk) after CIH (4 wk). B, The relative abundance of the 20 overlapping lncRNAs detected by Venn diagram in lncRNA array. n = 4 for each group. C, The expression of 17 out of the 20 overlapping lncRNAs was validated by qPCR analysis. Murine *GAPDH* gene was used as the housekeeping internal control. The transcript expression was quantified relative to the expression level of GAPDH using the comparative cycle threshold (ΔCt) method. Air indicates normoxia; Reo indicates reoxygenation after CIH; 4W indicates 4 wk; and 8W indicates 8 wk. n = 3/group. *P < .05. Data are presented as mean ± SEM

Interestingly, 20 lncRNAs simultaneously varied in both the MI‐CIH and MI‐reoxygenation groups, irrespective of CIH duration for 4 or 8 weeks (Figure 3A). The relative expression of these core 20 lncRNAs is shown in Figure 3B. For example, 16 lncRNAs, including *NONMMUT032513*, *NONMMUT074571* and *NONMMUT033183,* were elevated in MI‐CIH that recovered to control levels (MI‐Air) post‐reoxygenation, whereas two lncRNAs that decreased due to CIH returned to the control rates after reoxygenation. Furthermore, qRT‐PCR confirmed that the expression of *NONMMUT032513* and *NONMMUT074571* increased significantly (Figure 3C). It is interesting that *NONMMUT032513* and *NONMMUT074571* were not modulated by CIH in sham mice, indicating an alteration exclusively in MI conditions (Figure S4). Hence, CIH‐associated *NONMMUT032513* and *NONMMUT074571* might be the key lncRNAs in the aggravation of MI by CIH.

### 3.3 GO functional annotation and KEGG pathway analysis of CIH‐associated mRNAs

**Figure 4:**
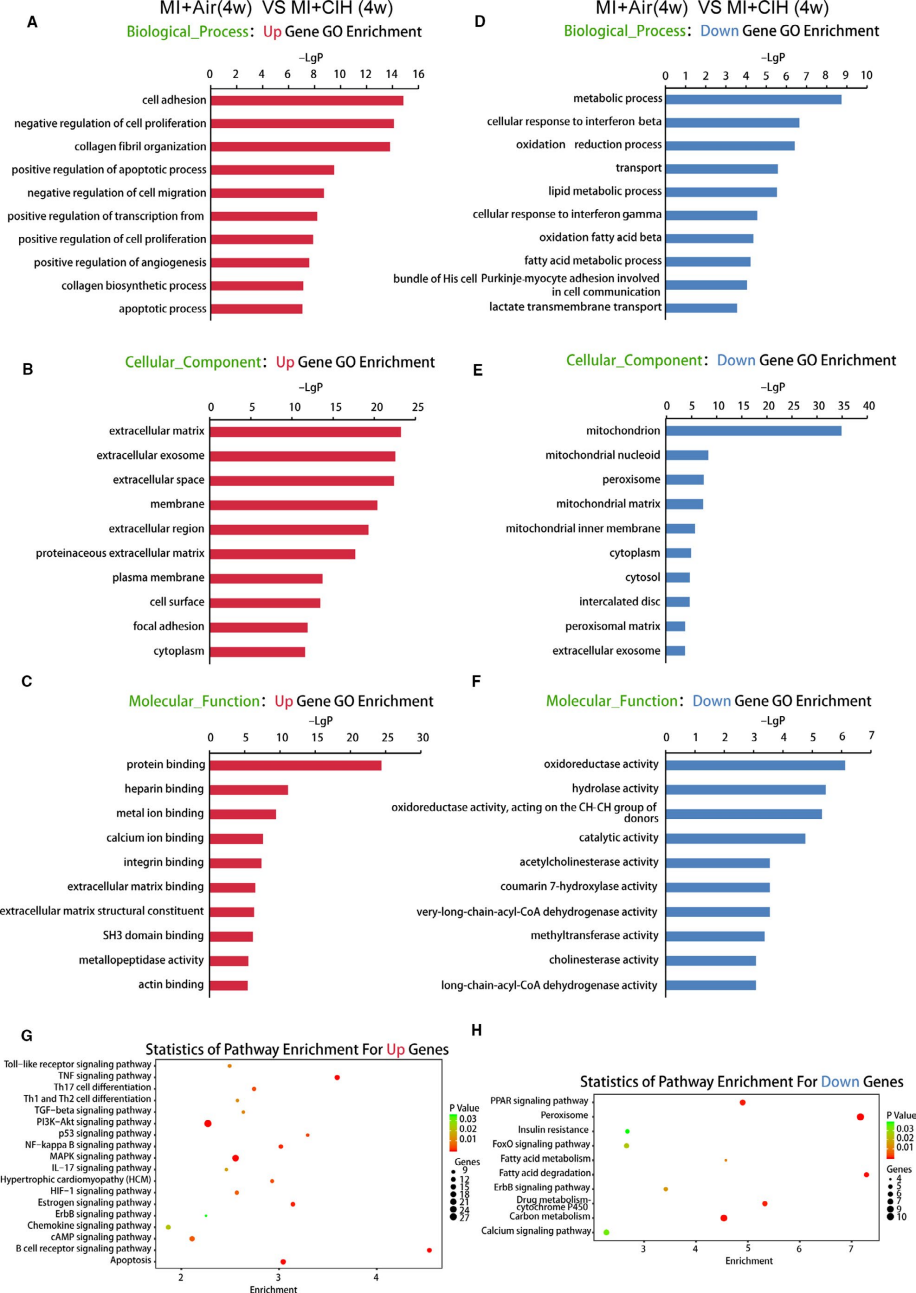
GO and KEGG pathway analysis for the mRNAs regulated by 4‐wk CIH. A‐C, Data show bioinformatics analysis of the GO terms in biological process, cellular component and molecular function of RNAs enriched in MI with 4 wk of CIH as compared to MI mice with air. D‐F, The GO terms that were deficient in MI with 4 wk CIH. G and H, KEGG pathways that were statistically different between MI with 4 wk CIH and MI with air. n = 4/group

In order to predict the potential functions of the CIH‐associated mRNAs identified in this study, GO and KEGG pathway analyses were carried out. In the biological process domain, functions including positive regulation of transcription from the RNA polymerase II promoter and collagen biosynthesis were up‐regulated in the MI‐CIH group and gene functions related to the cellular response to interferon beta, lipid metabolism and fatty acid metabolism were decreased (Figure 4 and Figure S5). The analysis of cellular component functions suggested that the extracellular exosome, extracellular space and extracellular regions were enhanced by CIH during MI injury, whereas the functions in the cytosol and cytoplasm were suppressed. Within molecular functions, aberrantly expressed mRNAs were involved in the up‐regulated GO function, including protein‐binding and down‐regulated GO function, such as hydrolase activity. For the KEGG pathway, CIH‐altered mRNAs were involved in the up‐regulated pathways, including Toll‐like receptor and Wnt signalling pathways and down‐regulated pathways, such as the PPAR signalling pathway.

Intriguingly, the analysis of the biological process gene functions was altered after reoxygenation showed that antigen processing and MHC presentation in addition to protein heterotetramerization were enhanced, whereas steroid and cholesterol biosynthesis were decreased. GO terms related to cellular components, including increased activity in extracellular regions and nucleosomes in MI mice after reoxygenation and reduction in membrane functions. Molecular functions, including DNA binding and transcription factor activities, were enriched and extracellular matrix binding was deficient in the reoxygenation group (Figure S6). Also, KEGG pathways for Th1, Th2 and Th17 cell differentiation were enriched, and steroid biosynthesis was reduced in the reoxygenation group.

### 3.4 lncRNA‐mRNA expression correlation network and target pathway

**Figure 5:**
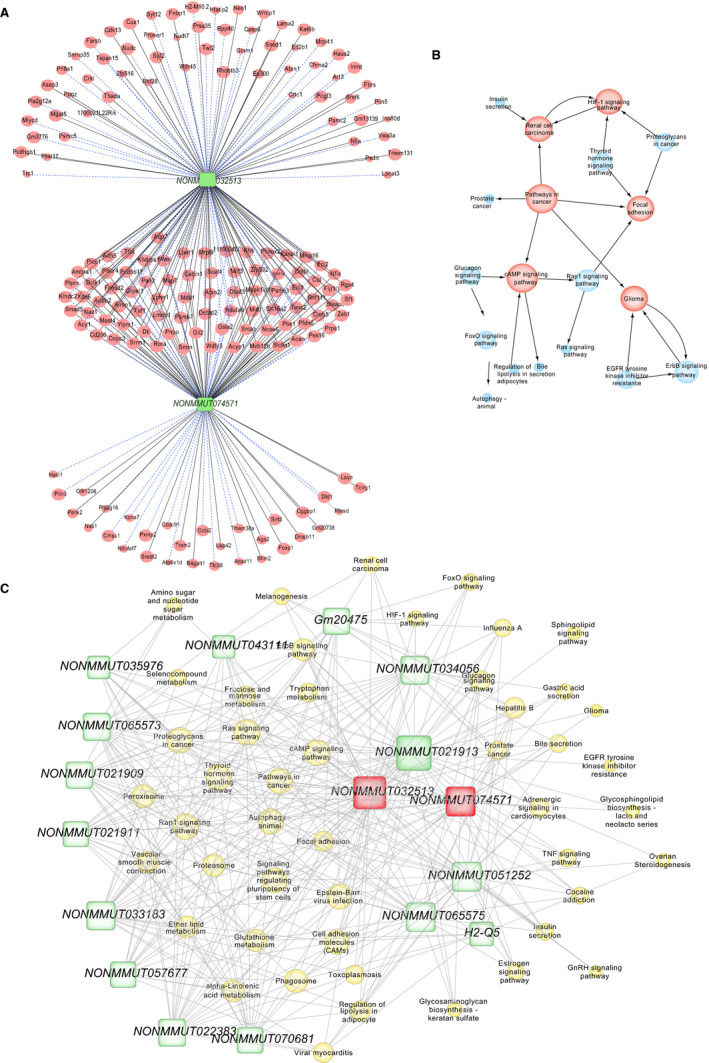
lncRNA‐mRNA co‐expression correlation network and target pathway network of core lncRNAs. A, LncRNA‐mRNA expression correlation network analysis of core lncRNAs (*NONMMUT032513* and *NONMMUT074571*) and their correlated mRNAs regulated by CIH post‐MI. Red nodes represent mRNAs, green blocks represent lncRNAs, and node area represents the value of betweenness centrality. The lines between nodes indicate a correlation, with a solid line representing positive correlation and a blue dotted line representing a negative correlation. B, Network of KEGG pathways associated with the core lncRNA‐linked mRNA. Red nodes represent core pathways, and node area represents the associated gene number, whereas lines represent an interaction between the pathways. C, Network of the core lncRNAs and target pathways. Yellow nodes represent pathways, green blocks represent lncRNAs, and node area represents the value of betweenness centrality. The lines between the nodes indicate a correlation. n = 4/group

The correlation and co‐expression network of lncRNA and mRNA was established to identify the possible linkage of lncRNA and downstream mRNA in MI‐CIH heart tissue (Tables S2,S3 and Figure S7). For the core lncRNAs *NONMMUT032513* and *NONMMUT074571*, we observed that lncRNA *NONMMUT032513* is positively linked with zinc finger E‐box binding homeobox 1 (ZEB1) and smad5, lncRNA *NONMMUT074571* is positively correlated with ZEB1 and Smtn, lncRNA *NONMMUT032513* is negatively associated with Cmbl and ADH5, and lncRNA *NONMMUT074571* is negatively correlated with Cmbl and Pfdn6 (Figure 5A). These lncRNAs are connected indirectly by their corresponding target genes, leading to a complicated lncRNA‐mRNA interaction network that is significantly influenced by CIH during the development of MI‐induced heart injury.

The modifications in these CIH‐regulated mRNAs are associated with MI aggravation and result in global changes in the activity of several molecular pathways, including cAMP, HIF‐1 and ErbB signalling (Figure 5B). Therefore, the network and interaction of potential signalling pathways indirectly regulated by lncRNA and their upstream regulators were constructed (Figure 5C). Among these lncRNAs, *NONMMUT032513* and *NONMMUT074571* exhibited the largest interaction network with cAMP signalling, followed by ErbB signalling and finally HIF‐1 signalling. The cAMP signalling pathway and *NONMMUT032513* and *NONMMUT074571* lncRNAs are indicated as the putative players most likely to contribute to heart injury under CIH conditions.

## 4 DISCUSSION

Accumulating evidence demonstrated that OSA plays a crucial role in cardiovascular disease. Nevertheless, the underlying molecular mechanisms are yet unclear. In the present study, we linked MI and obstructive OSA in a mouse MI model under CIH conditions. The cardiac function of MI mice was depressed by CIH, and RNA expression profiles were significantly altered. Specific lncRNAs, including *NONMMUT032513* and *NONMMUT074571*, were sensitive to CIH and reoxygenation during MI. In addition, these genes affected associated mRNAs, which might further regulate the potential GO functions and KEGG pathways involved in MI. The current findings delineated key lncRNAs responsible for exacerbated MI and cardiac damage by CIH.

OSA is highly prevalent in patients suffering from cardiovascular disease and is associated with increased risk of subsequent cardiovascular events.^20^, ^21^, ^22^ MI and myocardial damage, clinically severe conditions that increase patient morbidity and mortality,^23^ have been shown to be exacerbated by OSA.^24^, ^25^ One of the major characteristics of sleep apnoea syndrome is CIH, which plays a critical role during and after cardiac injury in driving and aggravating the pathophysiology of MI, including enhanced production of ROS in cardiomyocytes and activation of HIF‐1.^7^, ^8^, ^9^, ^10^, ^11^ These reports suggested a correlation between CIH and MI pathology. In this study, we confirmed the contribution of CIH to the outcome of MI in experimental animals. Both morphological changes, such as enlarged heart and infarct area and cardiac dysfunction such as decreased LVEF, FS and LVAW, were severe when exposed to 4‐week CIH after infarction. These observations were consistent with our recent findings.^19^ Following 8‐week CIH, the HW/BW ratios and cardiac function of MI mice have reached a threshold, with no difference between Air and CIH. On the other hand, CIH pre‐exposure to MI did not exert any significant effect on the severity of post‐MI remodelling or heart failure in comparison to the MI without CIH.^19^ Therefore, understanding the potential modulators mediating cardiac damage driven by CIH is necessary.

Currently, we are also exploring the mechanisms underlying CIH‐exacerbated post‐MI remodelling and have identified the key role of miR‐214‐3p in the suppression of cardiac CTRP9 expression, a novel cardioprotective cardiokine, contributing to CIH‐exacerbated cardiac remodelling.^19^ Recent reports highlighted the involvement of lncRNAs in the development of cardiovascular diseases, including hypertension,^26^, ^27^, ^28^ atherosclerosis^29^, ^30^ and atrial fibrillation.^31^, ^32^, ^33^ Also, the expression profiles of lncRNAs are affected by various factors, including hypoxia,^34^, ^35^, ^36^ energy stress^37^, ^38^, ^39^ and particulate matter.^40^, ^41^ Moreover, the impact of CIH on the expression profiles of lncRNAs and mRNAs in the heart samples of rat model has been examined previously.^42^ A total of 289 dysregulated lncRNAs were identified following 8 weeks of CIH, with a majority of novel lncRNAs, whose functions have not yet been elucidated. Herein, we showed that the expression of multiple lncRNAs was altered drastically after exposure to CIH for 8 weeks, whereas in the MI mice, 1482 lncRNAs were altered following CIH treatment (1235 increased and 247 decreased). Thus, CIH may exert a crucial role in driving cardiac injury in the presence of MI through modulating various lncRNAs. Herein, we focused on key lncRNAs, such as *NONMMUT032513* and *NONMMUT074571*, which were affected by CIH and restored to the baseline after reoxygenation. Thus, we suggested that these lncRNAs are sensitive to oxygen and hypoxia, and therefore may provide potential targets to prevent or ameliorate the adverse outcome of MI.

Further investigation based on the lncRNA‐mRNA expression correlation network analysis to predict the possible mechanism of these lncRNAs revealed that both *NONMMUT032513* and *NONMMUT074571* were significantly associated with genes involved in the pathology of MI. For instance, ZEB1, which is positively linked to *NONMMUT032513* and *NONMMUT074571* in our findings, has been identified as an extremely abundant protein in the infarct area of MI models.^43^ The high expression of ZEB1 promotes cardiac fibrosis, stabilizes collagen and aggravates MI by suppressing CXCR4 and increasing the level of collagen cross‐linking enzymes, Col1A1 and Col3A1.^44^, ^45^ Moreover, the clinical outcome after MI is highly correlated with NF‐κB, interleukin (IL)‐6 and IL‐8, which are direct targets of ZEB1.^46^, ^47^, ^48^ Cmbl is negatively associated with *NONMMUT032513* and *NONMMUT074571*, and implicated in converting the angiotensin II type I receptor antagonist, olmesartan medoxomil, to its bioactive metabolite olmesartan; also, it has been reported to be down‐regulated in the myocardial heart.^49^, ^50^ Therefore, it can be speculated that *NONMMUT032513* and *NONMMUT074571* responding to CIH might exacerbate the adverse outcome of MI by modulating the expression of the corresponding mRNAs, such as ZEB1 and Cmbl, which suppress the cardiac functions.

cAMP, ErbB and HIF‐1 signalling pathways are identified as potential targets of the core lncRNAs that are modulated by CIH. Both cAMP and ErbB signalling pathways were targets of *NONMMUT032513* and *NONMMUT074571*. Furthermore, enhanced ventricular G protein‐cAMP signalling has been detected under CIH exposure, which is associated with increased left ventricular contractility and augmented adrenergic activity in the cardiac tissue.^51^ In addition, the level of cAMP is also significantly high in the myocardial tissue of animals with chronic MI.^52^ Another study has shown that cAMP causes further ischemic myocardial damage in ischaemic heart failure by strengthening cell membrane Ca^2+^ permeability and increasing myocardial oxygen consumption.^53^ The ErbB signalling pathway is essential for cardiac development, and the decline in the level of ErbB plays a pathophysiological role in the development of cardiac dysfunction, leading to dilated cardiomyopathy.^54^, ^55^ The HIF‐1 signalling pathway, which is widely understood to be a hypoxia‐dependent pathway controlling the transcription of numerous genes involved in cardiovascular diseases, is suggested to be the potential target of *NONMMUT032513*. Moreover, it has been proposed that OSA activates the HIF‐1 pathway at the transcriptional level in a hypoxia dose‐dependent manner.^56^ Following MI, the activation of HIF‐1 is a leading factor in the hypoxic response and cardioprotective effect in the myocardium.^57^, ^58^

In conclusion, the lncRNA and mRNA expression profiles were screened in an experimental animal model, and the potential lncRNAs, critical for cardiac dysfunction following MI in response to CIH, were identified. The current data suggested that the hypoxia‐induced *NONMMUT032513* and *NONMMUT074571* modulate cAMP, HIF‐1 and ErbB signalling by targeting the corresponding mRNAs, such as ZEB1 and Cmbl. Further studies are required to confirm and identify the regulatory mechanisms among *NONMMUT032513* and *NONMMUT074571*, ZEB1 and Cmbl, and cAMP, HIF‐1 and ErbB signalling pathways.

## CONFLICTS OF INTEREST

The authors declare no conflicts of interest.

## AUTHOR CONTRIBUTION

**Chaowei Hu:** Conceptualization (equal); Data curation (equal); Formal analysis (equal); Funding acquisition (equal); Investigation (equal); Methodology (equal); Project administration (equal); Supervision (equal); Writing‐original draft (equal); Writing‐review & editing (equal). **Jing Li:** Data curation (equal); Funding acquisition (equal); Methodology (equal); Resources (equal); Software (equal); Writing‐original draft (equal); Writing‐review & editing (equal). **Yunhui Du:** Data curation (equal); Investigation (equal); Methodology (equal); Software (equal). **Juan Li:** Data curation (equal); Investigation (equal); Methodology (equal); Software (equal). **Yunyun Yang:** Data curation (equal); Investigation (equal); Methodology (equal); Software (equal). **Yifan Jia:** Investigation (equal); Methodology (equal); Software (equal). **Lu Peng:** Data curation (equal); Formal analysis (equal); Investigation (equal); Software (equal). **Yanwen Qin:** Conceptualization (lead); Funding acquisition (lead); Project administration (lead); Resources (equal); Supervision (equal); Writing‐original draft (equal); Writing‐review & editing (equal). **Yongxiang Wei:** Conceptualization (lead); Formal analysis (equal); Funding acquisition (lead); Project administration (lead); Resources (equal); Supervision (equal); Writing‐original draft (equal); Writing‐review & editing (equal).

## Supporting information

Fig S1‐S7

Table S1

Table S2‐S3

## Data Availability

Data sets used and analysed during the current study are available from the corresponding author on reasonable request.
