# Supplementary material for: Impact of chronic intermittent hypoxia on the long non‐coding RNA and mRNA expression profiles in myocardial infarction
Source: J Cell Mol Med. 2020 Nov 20;25(1):421–33. doi: 10.1111/jcmm.16097 (PMC7810970; doi:10.1111/jcmm.16097)
Supplement: Supplementary file 1 — Fig S1‐S7 [file JCMM-25-421-s001.doc]

**Supplementary material**

**
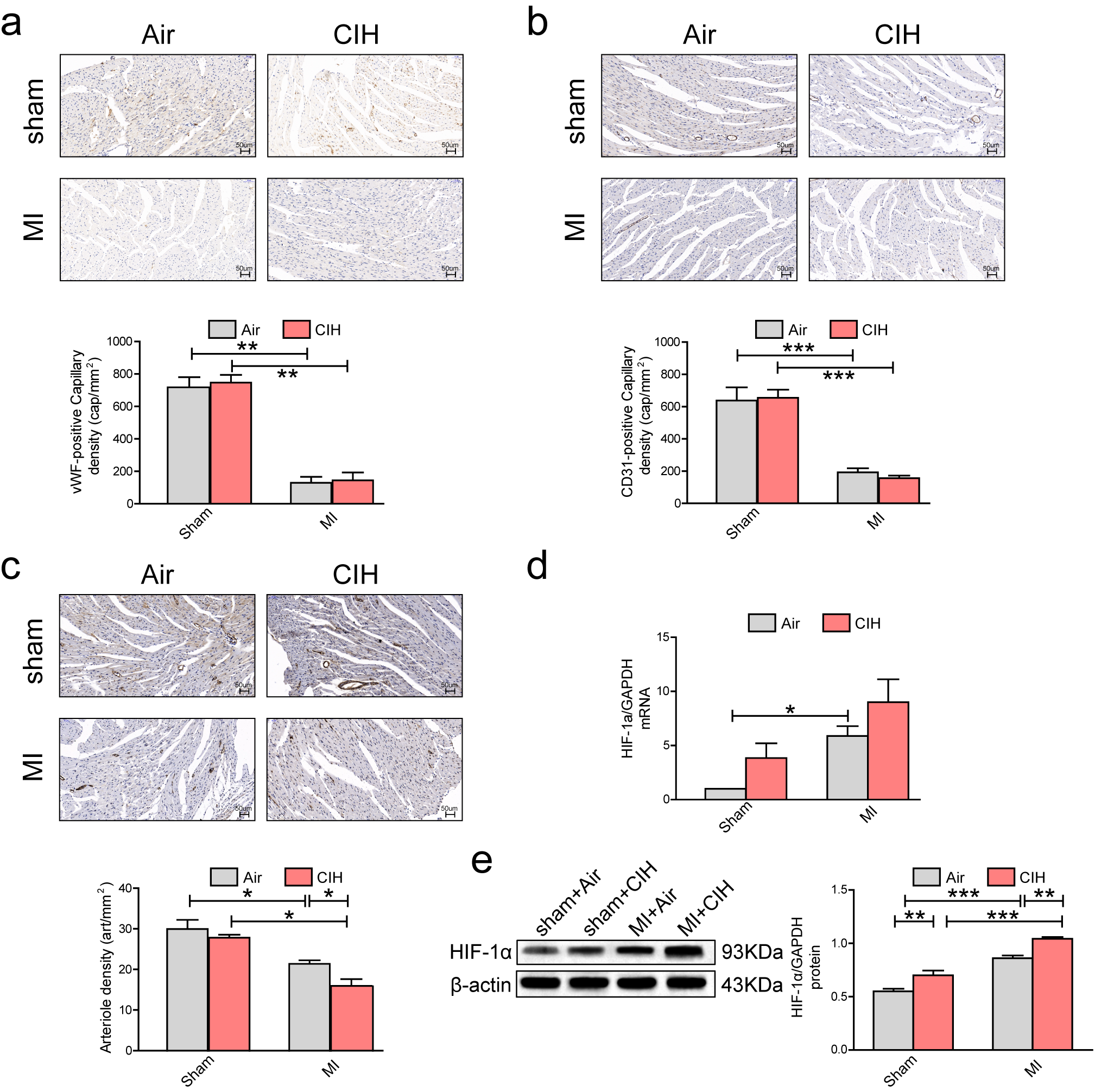
**

**Fig. S1. Myocardial capillary and arteriolar density in parallel to HIF-1α expression following CIH exposure.**

(a-c) Immunohistochemistry analysis of capillaries (vWF and CD31 marked, respectively) and arterioles (α-SMA marked) in infarcted hearts of MI mice under 4-weeks of CIH. n=4-5/group. Scale bars are 50μm. (d) The mRNA expression of myocardial HIF1-α following CIH treatment was validated by qPCR analysis. n=3 for each group. (e) Protein expression of myocardial HIF-1α following CIH treatment was validated by western blot analysis. n=4/group. Air indicates normoxia. **p*<0.05, ***p*<0.01, ****p*<0.001. Data are presented as mean ± sem.

**
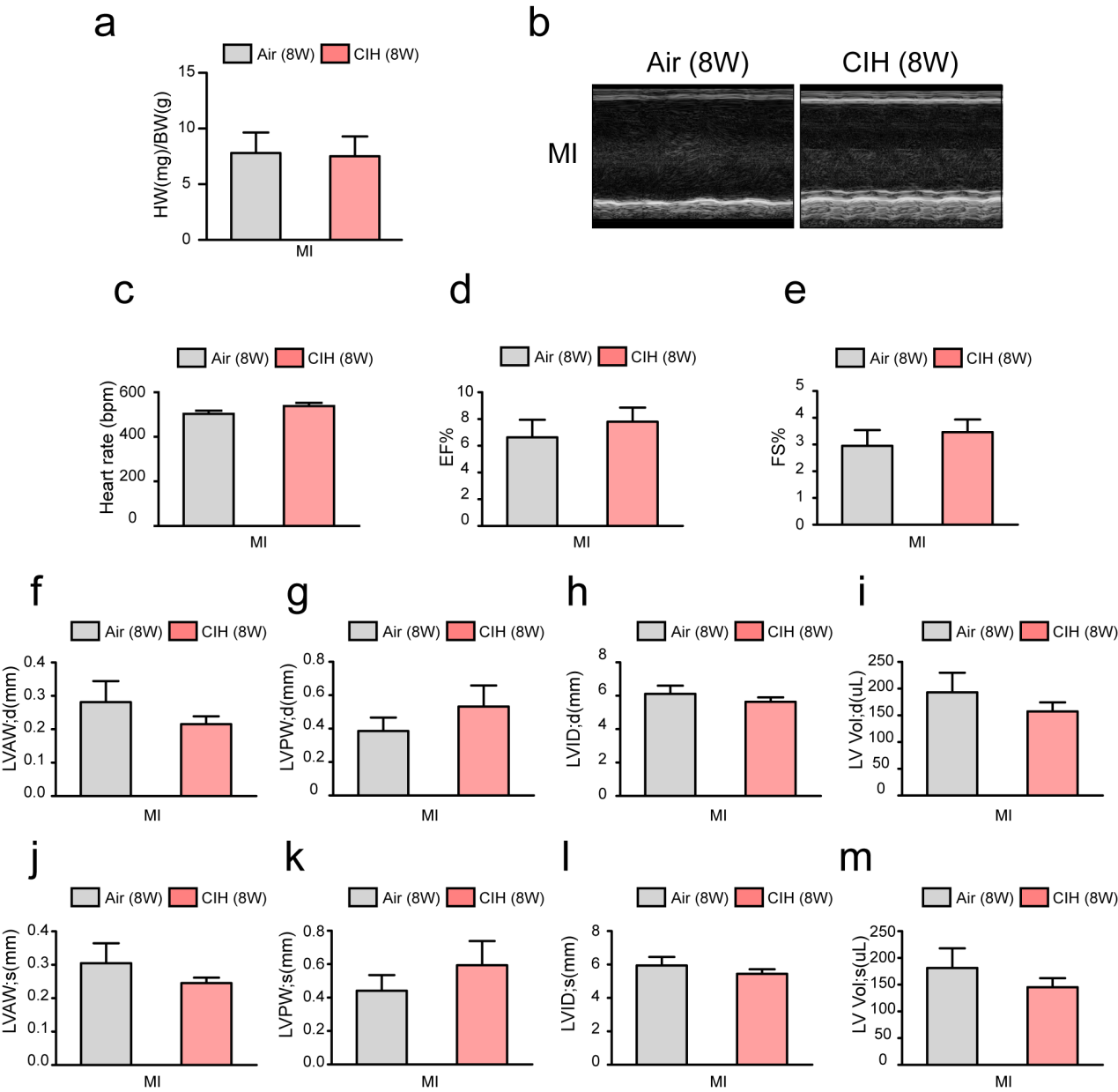
**

**Fig. S2. Cardiac injury of mice suffered 8-weeks CIH post-infarction.** (a) Heart weight/body weight ratio (HW/BW) of MI mice under CIH for 8 weeks. n=4/group. (b) Cardiac function was examined by echocardiography and representative left ventricular M-mode echocardiography images are shown. (c–m) Quantification of heart rate, EF, FS, LVAWd, LVAWs, LVPWd, LVPWs, LVIDd, LVIDs, LV Vol-d, LV Vol-s. Air indicates normoxia. n=4/group. Data are presented as mean ± sem.

**
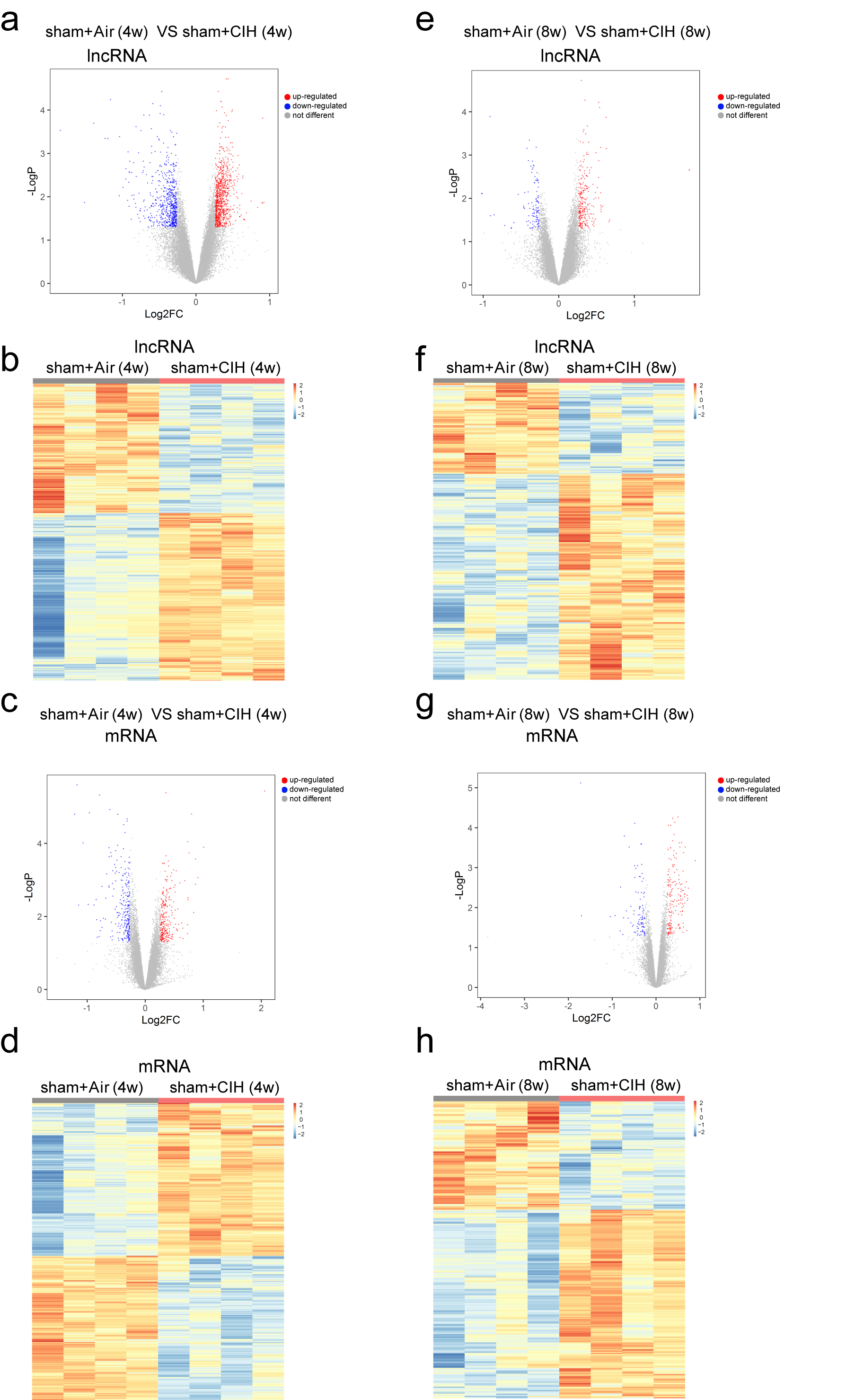
**

**Fig. S3. Specific lncRNAs and mRNAs regulated by CIH in sham mice.**

(a, c) Volcano plots showing the lncRNAs and mRNAs significantly regulated by 4-week exposure to CIH. The lncRNAs and mRNAs with *p*≤0.05 (t-test) and fold-change in expression ≥1.2 were regarded as differentially expressed between groups. (b, d) Heatmap depicting the changes in lncRNAs and mRNAs in MI mice after exposure to CIH for 4 weeks. (e, g) Volcano plots of the significantly different lncRNAs and mRNAs between sham and sham+CIH groups at 8 weeks. (f, h) Heatmap of the relative abundance of significantly changed lncRNAs and mRNAs by CIH at 8 weeks. Air indicates normoxia; 4W indicates 4 weeks; 8W indicates 8 weeks. n=4/group. The red scatters in volcano plots indicate genes upregulated by CIH, blue scatters indicate genes downregulated, and gray scatters indicate genes that were not different between the groups. The heatmap scale indicates the relative abundances of specific genes that were transformed into Z scores. The value of (-logP) was the base 10e negative logarithm of the *p*-value.

**
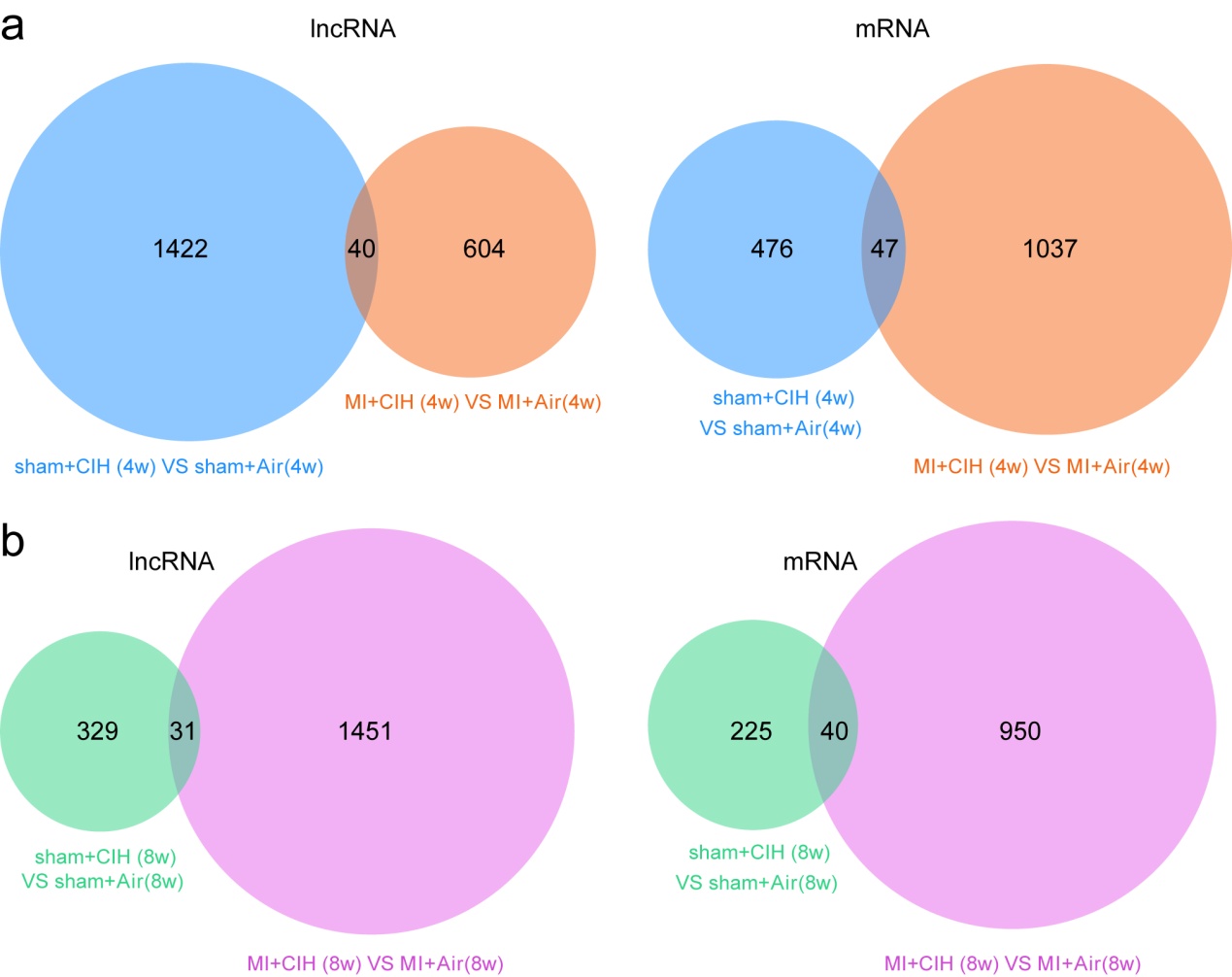
**

**Fig. S4. The number of shared and exclusively altered lncRNAs and mRNAs in response to CIH between sham and MI conditions.**

(a) Venn diagram showing the number of lncRNAs and mRNAs that are statistically altered by 4 weeks of CIH between sham and MI mice. (b) The overlap for the 8 weeks CIH-induced differential expression of lncRNAs and mRNAs between sham and MI conditions is shown in venn diagram.

**
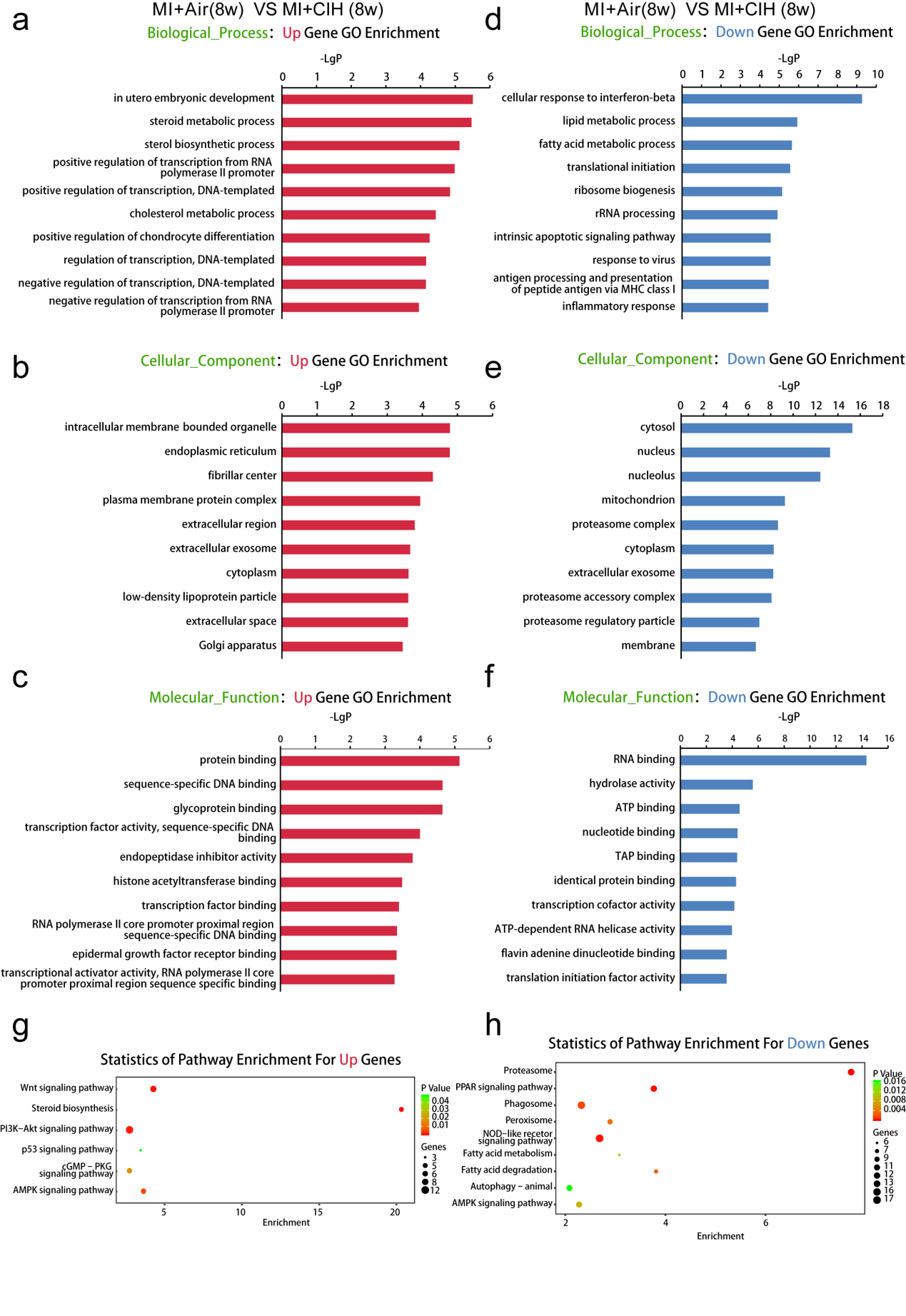
**

**Fig. S5. GO terms and KEGG pathways of the mRNAs regulated by 8-week CIH.**

(a–c) Data show bioinformatics analysis of the GO terms in biological process, cellular component, and molecular function form RNAs enriched in MI with 8 weeks of CIH as compared to MI mice with air. (d–f) GO terms that were deficient in MI with 8 weeks CIH. (g-h) KEGG pathways that were statistically different between MI with 8-weeks CIH and MI with air. n=4/group.

**
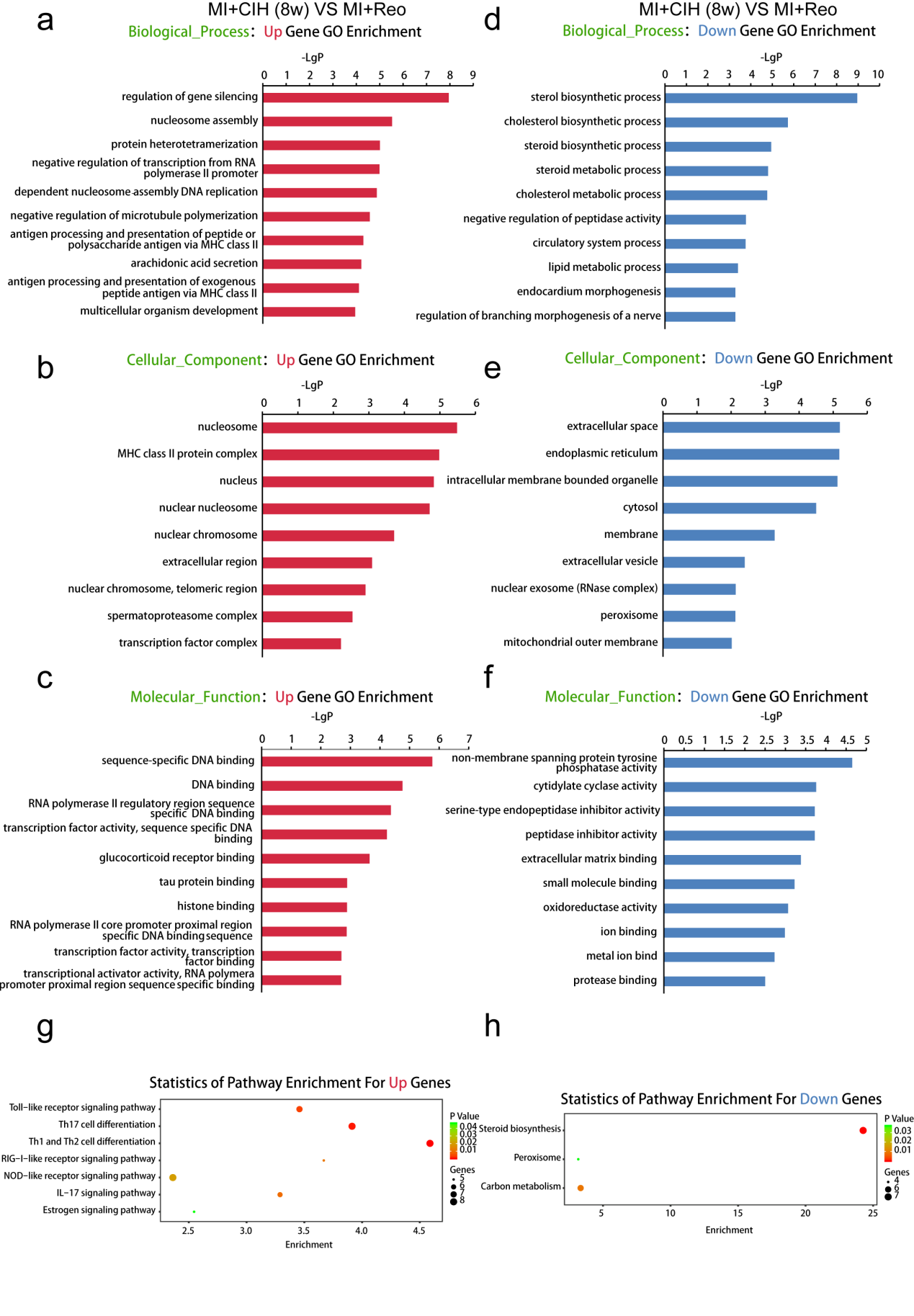
**

**Fig. S6. GO and KEGG pathway analysis for the mRNAsregulated byreoxygenation after CIH.**

(a–c) Data show bioinformatics analysis of the GOterms in biological process, cellular component,and molecular function formRNAsenriched in MI mice with reoxygenation (4 weeks) after CIH (4 weeks) as compared to MI mice with 8-weekCIH. (d–f) GOterms that were deficient in MI mice with reoxygenation after CIH. (g-h) KEGG pathways that are significantly different between MI with reoxygenation after CIH and MI with 8-weekCIH. n=4/group.


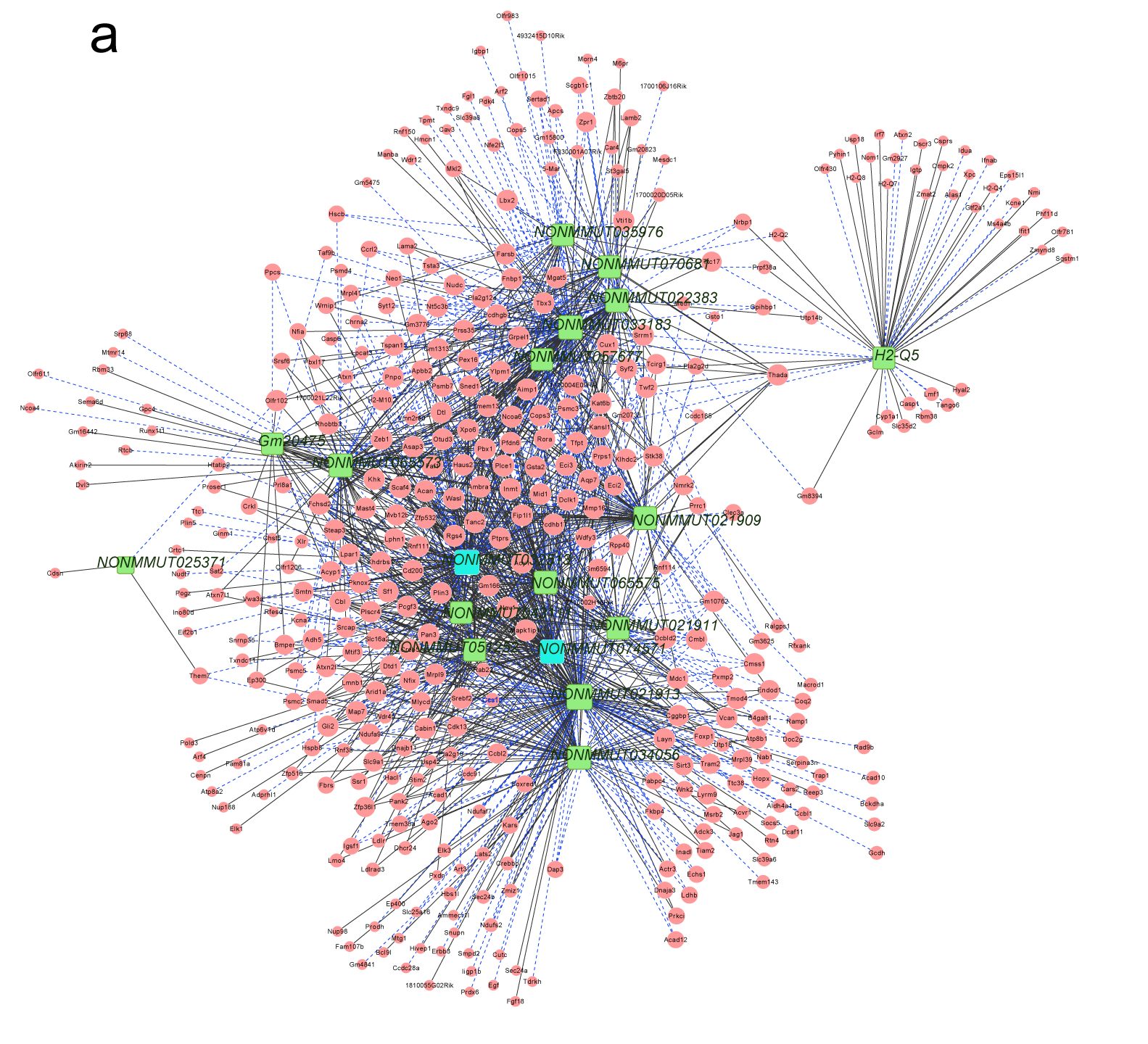


**Fig. S7. LncRNA-mRNA expression correlation network analysis of lncRNAs and their correlated mRNAs regulated by CIH post-MI.** Red nodes represent mRNAs, green blocks represent lncRNAs, and node area represents the value of betweenness centrality. The lines between nodes indicate a correlation, with a solid line representing positive correlation and a blue dotted line representing a negative correlation.
