# Supplementary material for: Impact of chronic intermittent hypoxia on the long non‐coding RNA and mRNA expression profiles in myocardial infarction
Source: J Cell Mol Med. 2020 Nov 20;25(1):421–33. doi: 10.1111/jcmm.16097 (PMC7810970; doi:10.1111/jcmm.16097)
Supplement: Supplementary file 2 — Table S1 [file JCMM-25-421-s002.doc]

**Table. S1** Primer Pairs

| **Gene** | **Primer** |
| --- | --- |
| NONMMUT035976_F | CTGGAGAGGTGGCTCATTGG |
| NONMMUT035976_R | ACCATGTGGTTGCTGGGAAT |
| NONMMUT051252_F | AGTGCACTAGAAGCTCCCCT |
| NONMMUT051252_R | TTCCAGTTCTCACGGTCTGC |
| Gm20475_F | CAGGTGTTGAGGGCGAAAGT |
| Gm20475_R | CACGCACACAATGTTTCCCA |
| NONMMUT057677_F3 | TCACCAGCTGAGGACCACTA |
| NONMMUT057677_R3 | GGCCTCGAAGCTCAACTCTT |
| NONMMUT043111_F | ACTGAAATGTGAGGGGGCTG |
| NONMMUT043111_R | ACAGTTGTCAGACACGCCAT |
| NONMMUT065573_F | CCCAGACTTGAGTTGCGAGT |
| NONMMUT065573_R | TCCACTCCAGGACCCAAGAA |
| NONMMUT065575_F | GGGCGTTCCCAACAAATGAC |
| NONMMUT065575_R | CCAACAAAACGCGTGGGTAG |
| NONMMUT070681_F | TCCCTGGTGCAGTTAGGAGT |
| NONMMUT070681_R | AGTGAGTTCTCCCACCAATTCC |
| NONMMUT074571_F | ACAGAAAATGGGGGCATGGT |
| NONMMUT074571_R | ACCACGTCCCAGCGTAAAAT |
| NONMMUT022383_F | ATGTGACTTCCCAGCCAAGG |
| NONMMUT022383_R | TGGGAAGGATCAGAAACCGC |
| NONMMUT021909_F | ATCATACCAGCAACCGCTCT |
| NONMMUT021909_R | ATGACTCCACGCTGGAAAGG |
| NONMMUT021911_F2 | CTTGTGAGACTATGCCGGGG |
| NONMMUT021911_R2 | GGCCGACTAGGCCATCTTTT |
| NONMMUT021913_F | ACCCTGCAGGCCTGATTTAC |
| NONMMUT021913_R | TTCACAGCCCCCTGACATTC |
| H2-Q5_F | GCAGGCTGGTATTGCAGAGA |
| H2-Q5_R | ACTGCCAAGTCAGGGTGATG |
| NONMMUT032513_F | CAGAGGCCCCTCTTATCCGA |
| NONMMUT032513_R | ATGTCATCGGAGCTGTGGTC |
| NONMMUT033183_F | AGACCGTGATAGGCTCCCTT |
| NONMMUT033183_R | CGGAATTCTTGACACACGCC |
| NONMMUT034056_F4 | TCCTGTGGTGTCCCTATCCC |
| NONMMUT034056_R4 | CATGGGCAAGCACCAATTCC |
| HIF-1α_F | CGACACCATCATCTCTCTGG |
| HIF-1α_R | AACCTCTTGATTCAGTGCAG |
| Mus GAPDH-F | ATCATCCCTGCATCCACTGG |
| Mus GAPDH-R | TGCCTGCTTCACCACCTTCT |
